# Using 21st-Century Technologies to Determine the Cognitive Capabilities of a 11,000-Year-Old Perak Man Who Had Brachymesophalangia Type A2

**DOI:** 10.21315/mjms2021.28.1.1

**Published:** 2021-02-24

**Authors:** Johari Yap Abdullah, Mokhtar Saidin, Zainul Ahmad Rajion, Helmi Hadi, Norshahidan Mohamad, Cicero Moraes, Jafri Malin Abdullah

**Affiliations:** 1 Craniofacial Imaging Laboratory, School of Dental Sciences, Universiti Sains Malaysia, Kelantan, Malaysia; 2 Center for Global Archaeological Research, Universiti Sains Malaysia, Pulau Pinang, Malaysia; 3 Kulliyyah of Dentistry, IIUM Kuantan Campus, Kuantan, Pahang, Malaysia; 4 School of Health Sciences, Universiti Sains Malaysia, Kelantan, Malaysia; 5 School of the Arts, Universiti Sains Malaysia, Pulau Pinang, Malaysia; 6 Ortogonline Treinamento em Desenvolvimento Profissional e Consultoria Ltda, Sinop, MT, Brazil; 7 Department of Neurosciences, School of Medical Sciences, Universiti Sains Malaysia, Kelantan, Malaysia; 8 Brain and Behaviour Cluster, School of Medical Sciences, Universiti Sains Malaysia, Kelantan, Malaysia; 9 Department of Neurosciences and Brain Behaviour Cluster, Hospital Universiti Sains Malaysia, Universiti Sains Malaysia, Kelantan, Malaysia

**Keywords:** 3D reconstruction, forensic facial reconstruction, cognitive function, computer-aided design, neuroanthropology

## Abstract

Perak Man, named after the state where the skeleton was found, was the most complete skeleton found in Southeast Asia. The funerary artefacts indicate that Perak Man was highly respected, as he was buried at the centre of the highest cave in Lenggong, and he was the only person buried there. A copy of the original skull was made using computed tomography (CT) and 3D printing. Based on the internal structure of the reconstructed skull, the estimated intracranial volume (ICV) is 1,204.91 mL. The hypothetical face of Perak Man was reconstructed according to established forensic methods. Based on his presumed status, Perak Man was likely a respected person in the group and, perhaps, a shaman and the most knowledgeable person in the group regarding survival, hunting, gathering and other aspects of Palaeolithic daily life.

## Introduction

**Figure 1:**
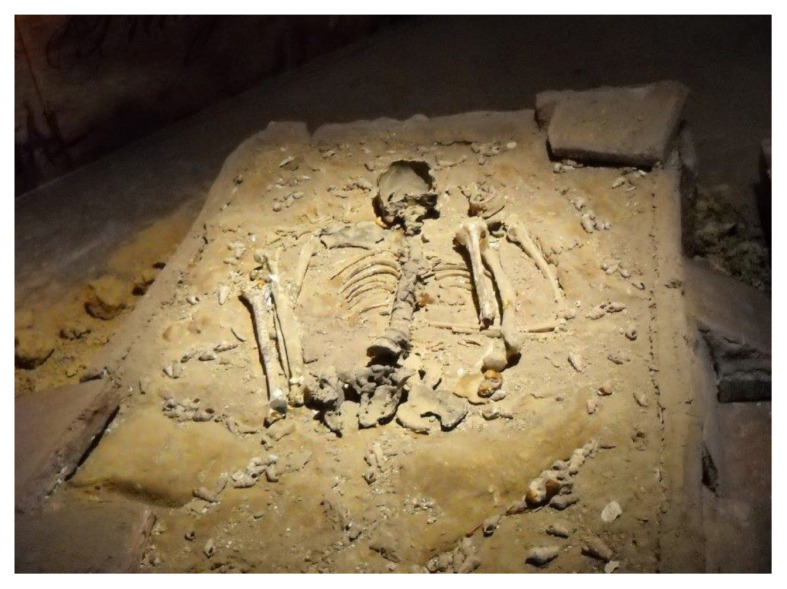
Perak Man skeleton, exhibited in the Lenggong Archaeological Gallery, Perak

The excavation of Gua Runtuh in Lenggong Valley, Perak, Malaysia in 1990 revealed a 10,000–11,000-year-old primary burial site of an adult human buried in the foetal position (1) as shown in Figure 1. He was named Perak Man after the state where his skeleton was found and was the most complete skeleton found in Southeast Asia from the antiquity range, which is 10,000–11,000 years before the present. Lenggong Valley was also recognised as a World Heritage Site by the United Nations Educational, Scientific and Cultural Organisation (UNESCO) (2).

Based on Jacob and Soepriyo’s study (3), Perak Man was a male, as indicated by the pelvic bones, sacrum, skull and femora. Based on other skulls found and traits observed in the skull, humeri, femora and tibiae suggest Australo-Melanesoid ancestry. Perak Man is estimated to have been about 154 cm tall, and based on the cranial suture closure, lipping of the vertebral bodies, and scapula and the pelvic bones, thinness of the scapula, and degree of tooth wear, he was about 40–45 years old, at death which is considered an old age for that period.

The funerary artefacts indicate that Perak Man was highly respected, as he was buried at the centre of the highest cave in Lenggong, and he was the only person buried there. Ten stone artefacts (two hammerstones, two slabs, five oval unifacial pebble tools and one miscellaneous tool) were found, along with 2,878 riverine shells and animals’ bone remains weighed 1.261 kg (1).

As a large proportion of Perak Man’s skull was still intact, we should be able to get more information about his life and the life of his society at that time. However, since the skull was more than 10,000 years old, it was too fragile to be handled freely. Thus, additive manufacturing technology using 3D printing was applied to create an accurate life-size model of the skull from the captured computed tomography (CT) data (4). This technology has been widely utilised by many archaeological teams around the world to build models of excavated remains.

The greatest advantage of using this technology is that members of multidisciplinary archaeological teams are no longer required to gather at the site of discovery, which is typically remote. They can now contribute their efforts and expertise while remaining in their home countries, as the archaeological models can be sent to them for their expert opinion. Therefore, although using additive manufacturing technology for archaeological 3D modelling is expensive, the overall research costs are much cheaper (4).

A copy of the original skull was made by CT and 3D printing. The face was reconstructed according to established forensic methods. These methods are based on the average tissue thicknesses measured at various landmarks on the skull, and guidelines are used for the shape, size, and position of different facial features. These guidelines were applied to the unique anatomy and measurements of the chosen skull.

The traditional facial reconstruction methods were based on manual procedures and use 3D sculptures made from a 3D model of the skull (5). Some anatomical landmarks were set on the raw skull and then the average soft tissue thickness was applied to estimate a corresponding landmark on the face. Finally, the face was sculpted based on the estimated landmarks (6). However, the accuracy of clay-based sculpting depends on the ability of the operator (7) and is a highly subjective procedure; it requires a great deal of anatomical and artistic modelling expertise (8) and is labour intensive (9).

## A Similar Archaeological Finding

A similar archaeological finding was published by National Geographic on 4 June 2020 by Jaen (10). A team of archaeologists ventured into a cave in Gibraltar, where they found a burial containing bones and a skull. The skull was partially damaged after burial and has been kept at the Gibraltar National Museum. However, the skull’s age remained a mystery for many years. In 2019, the results of a landmark study proved through DNA analysis that it belonged to a woman who lived 7,500 years ago, making it the oldest remnant of a modern female woman found in Gibraltar to date.

The Gibraltar National Museum team worked to create a forensic facial reconstruction of her face from the skull. The skull was scanned and computer-aided design was used to recreate missing and damaged parts, including the mandible. They spent 6 months creating her lifelike appearance using clay and, later, using silicone on the 3D printed model. She was named Calpeia by the researchers, who were inspired by the classical term for Gibraltar, known in ancient times as Mons Calpe (11).

Reconstructing the 3D facial model of an unidentified individual from his or her skull provides significant benefits in terms of archaeology, anthropology and forensic investigation, but the process was complicated. Using computer-aided design to reconstruct an individual’s 3D facial features based on a skull or part of a skull has the significant advantage of reducing the amount of time required to complete the process.

## 3D Virtual Reconstruction of Perak Man

**Figure 2:**
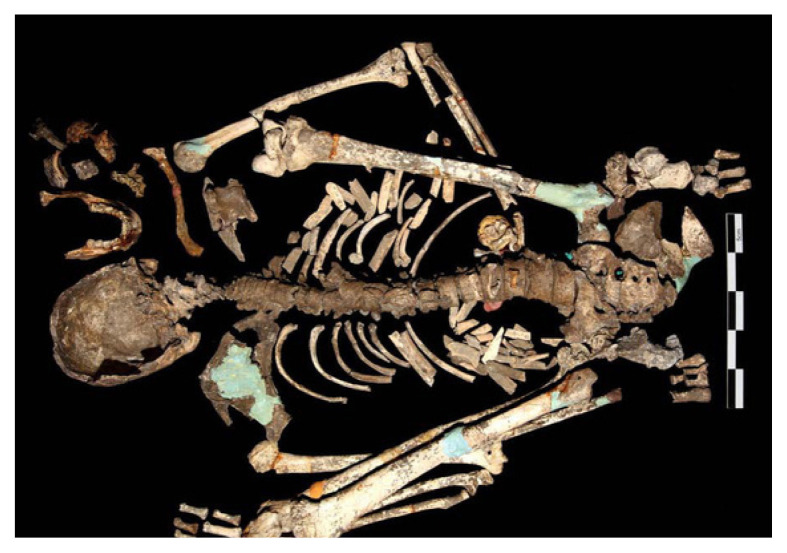
Fragments of Perak Man’s skull and skeleton

We chose to perform a similar facial reconstruction of Perak Man but realised that the process might be a bit more complicated since the skull bones were in fragments: the posterior portion in articulation, comprising the occipital, parts of both parietals and parts of both temporal bones; a fragment of the left side of the frontal bone; three fragments of the parietal bones; fragments of both zygomatic bones; maxillary fragments with three teeth; the mandible, incomplete, with a fragment of the left condylar process, as shown in Figure 2.

The skull was scanned using CT in the Radiology Department of the Hospital Universiti Sains Malaysia using a Light Speed Plus scanner (General Electric Medical System, US) with a 1.25 mm section thickness in spiral mode and a 512 × 512 matrix. Scanning was performed with a tube current of 200 mA at a 120 KVP. The resulting images were stored in Digital Imaging and Communications in Medicine (DICOM) format. Segmentation of the images was performed using Mimics v17.0 software (Materialise NV, Heverlee, Belgium) and exported in Standard Tessellation Language (STL) format for the 3D reconstruction process.

**Figure 3:**
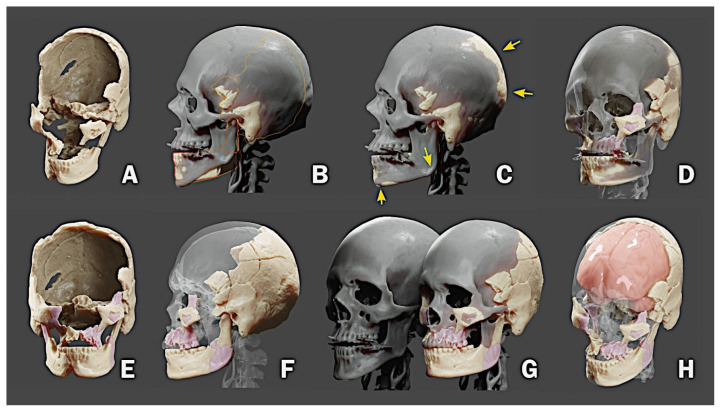
The skull reconstruction process

The skull of Perak Man comprised of fragments that only accounted for a small region of the entire structure [A], as shown in Figure 3. Due to the characteristic of the bones, it was possible to position them in three-dimensional space, fitting broken parts or accommodating the missing volume using the skull anatomy as a reference. To facilitate the process, a 3D mesh from a virtual donor was used (i.e. a real skull was used to help position the pieces in 3D space). However, the skull structure of the virtual donor often differed from the one that is being reconstructed [B].

Blender 3D modeling and animation software (blender.org) provides a series of tools for editing three-dimensional objects. It is possible, with this software, to deform specific parts of an object to make it compatible with another. In the case of the skull, this work was done with various tools that stretched and pushed parts of the mesh while keeping others intact. Initially, we sought to deform the skull of the virtual donor to make it compatible with that of Perak Man [C].

The donor’s skull became a reference for the movement of the pieces, eventually indicating that one or another original piece was not in the correct location. For the reconstruction to be as consistent as possible with the original pieces, some of them were copied and mirrored to enlarge the region of the original structure [D, E]. After a series of deformations and adjustments, a structural approximation of what would be the skull in its original state [F] was obtained. At the end of the process, there was a big difference between the skull of the virtual donor and that of Perak Man [G]. Based on the internal structure of the reconstructed skull, it was possible to calculate the volume corresponding to the brain mass [H]. The estimated intracranial volume (ICV) was 1,204.91 mL.

## Forensic Facial Reconstruction

**Figure 4:**
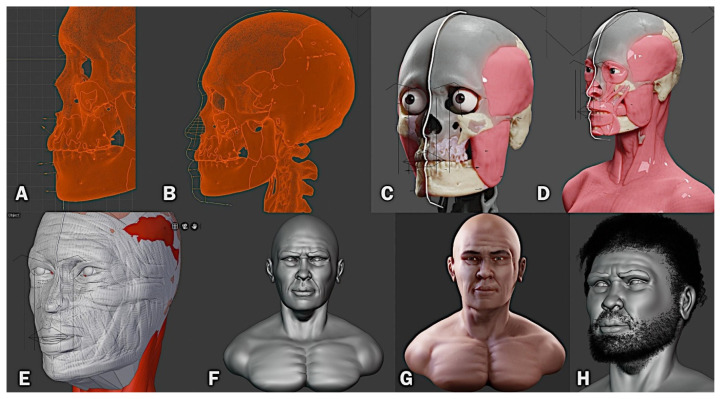
Forensic facial reconstruction process used on Perak Man

Forensic facial reconstruction is a technique that reconstructs the facial features of a skull for the purpose of individual recognition (12). The forensic add-on for Blender is a set of commands arranged sequentially, which allowed the user to proceed with the digital forensic facial reconstruction process within Blender 3D software. It is a solution based on free software, openly available for the three most popular operating systems on the market: Windows, Linux, and Mac OSX (13), as shown in Figure 4.

After the skull was reconstructed, it was positioned on the plane of Frankfurt and a series of soft tissue thickness markers were placed based on two in vivo studies carried out in an Asian population; both [A] central (14) and symmetric markers (15) were replaced. Next, the nose projection [B] was traced using two approaches as a reference: the Russian method and the Manchester method. The joint process was described in detail by Moraes and Miamoto (pp. 338–352) (16). Using the markers distributed on the skull and the projection of the nose as a limit reference, the profile of the face was then drawn. An orbit was imported, anatomically positioned and edited according to the individual’s ancestral characteristics. Facial muscles were imported and adapted to the structure of the skull: temporal, masseter, orbicularis eye, upper lip lift, nasal, zygomatic, orbicularis lip, buccinator, mentalis, sternocleidomastoid and others. Complementary information was also drawn, such as the frontal design of the lips and eyebrows [C, D].

**Figure 5:**
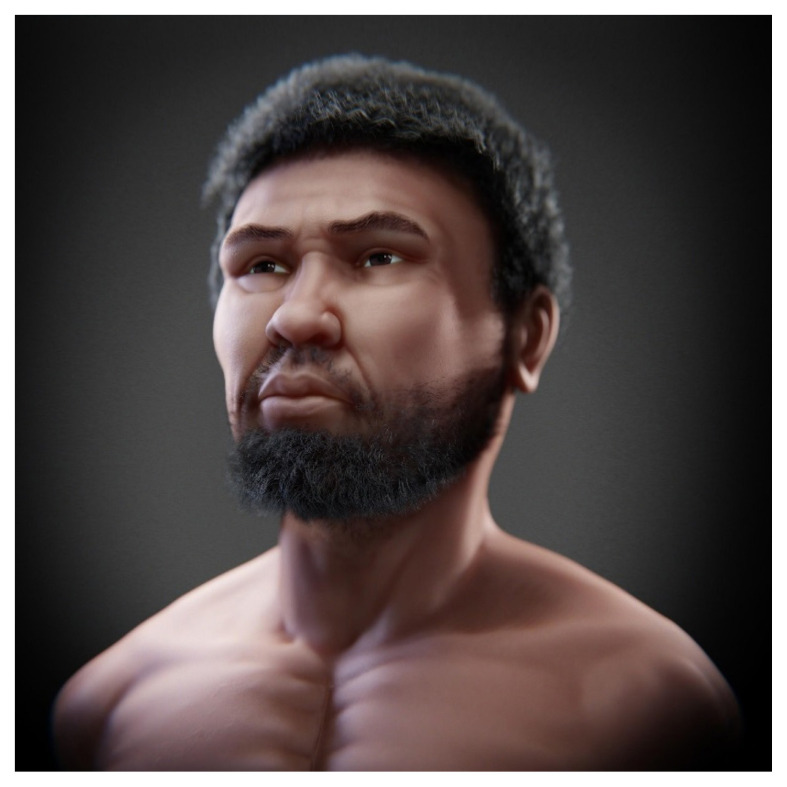
The hypothetical face of Perak Man

A low-resolution digital sculpture was made to adapt it to the volume indicated by the markers and facial traces [E]. This sculpture was then improved gradually by increasing the structural details until the process was completed [F]. The mesh from the digital sculpture was simplified using the ‘retopology’ process (17), followed by the digital painting process [G]. Following anthropological guidelines, the hair and beard were shaped [H]. Finally, a high-resolution image was generated, as shown in Figure 5.

In this study, the estimated volume of Perak Man’s ICV was 1,204.91 mL, which was smaller than modern humans’ cranial capacity, as shown in Table 1.

A study conducted by Musa (18) among the Malay population in Kelantan reported the mean ICV as 1,375 (148.6) mL based on the measurements of a total of 59 subjects (27 males and 31 females aged 15–50 years old). A study performed by Embong et al. (19) of the normal Malay population found similar results, with a mean ICV value of 1,332.5 (142.9) mL based on the measurements of 43 subjects (19 males and 24 females aged 50–77 years old). Similar studies undertaken in Turkey (20), the United Kingdom (UK) (21, 22), France (23), the United States (US) (24) and Germany (25) revealed that their ICV was larger than that of Perak Man. A study conducted by Riello et al. (26), which categorised subjects by gender and age, found that the mean ICV of 38 males between the ages of 40 years old and 60 years old was 1,469 mL.

Despite being born with a physical handicap, Perak Man survived and lived a nomadic hunting and gathering lifestyle, probably due to considerable care given to him. He may have overcame his physical handicap by developing proficient hunting skills, or perhaps he rarely hunted and took on other tasks. Based on his presumed status, Perak Man was likely a respected person in the group and, perhaps, a shaman and the most knowledgeable person in the group regarding survival, hunting, gathering, and other aspects of Palaeolithic daily life (1, 27).

Perak Man likely lived as a hunter, similar to the lifestyle of the Negrito ethnic group, which could be the descendant of Perak Man. In Perak Man’s time, they lived in an area with many caves, such as Badak Cave, Teluk Kelawar Cave, Harimau Cave, Gelok Cave, Batu Puteh Cave and Kajang Cave; based on drawings in the caves, they used the caves as temporary camps. The drawings revealed that their way of life included hunting, collecting food, catching fish and gardening (28).

## Conclusion

This neuroanthropological finding of Perak Man will path the way towards:

- Use 21st-century technology to understand how interactions between the brain, mind, behaviour and culture occurred in the past.
- Examine the role of the nervous system in the creation of social structures in both the past and the present.
- Conduct empirical and critical inquiries into the incorporation of previous, current and future findings into modern neurosciences and hypothetical anthropological ideologies.
- Use the neuroanthropological sciences to improve our understanding of human science theories across the world.

## Figures and Tables

**Table 1:** Comparison of ICV from this study against other published data

	Location	Modality	ICV (mL) SD	*N*
Perak Man	Malaysia	CT	1,204.91 (N/A)	1
Asian population				
Embong et al. (19)	Malaysia	MRI	1,332.5 (142.9)	43
Musa (18)	Malaysia	MRI	1,375.0 (148.6)	59
Acer et al. (20)	Turkey	CT	1,312.0 (133.1)	28
Western population				
Whitwell et al. (22)	UK	MRI	1,374.0 (150.0)	51
Ricard et al. (23)	France	CT	1,384.6 (135.3)	58
Nandigam et al. (24)	US	MRI	1,442.8 (116.6)	47
Riello et al. (26)	Italy	MRI	1,469.0 (126.0)	38
Strik et al. (25)	Germany	CT	1,424.0 (145.7)	12
Jenkins et al. (21)	UK	MRI	1,407.1 (148.9)	52
